# Supplementary material for: In Vivo Validation of Novel Synthetic tbp1 Peptide-Based Vaccine Candidates against Haemophilus influenzae Strains in BALB/c Mice
Source: Vaccines (Basel). 2023 Oct 27;11(11):1651. doi: 10.3390/vaccines11111651 (PMC10675187; doi:10.3390/vaccines11111651)
Supplement: Supplementary file 1 [file vaccines-11-01651-s001.zip › vaccines-2555674-supplementary.pdf]

In-vivo Validation of Novel Synthetic tbp1 Peptide-Based Vaccine Candidates against Haemophilus influenzae Strains in BALB/c Mice

Supplementary Data

Supplementary Table S1. Summary of Physical and Chemical Analysis Results (Bibi et al., 2021) [35]

	Pal omp6	Gdh	Tbp1	PilW typeIV	Porin OmpA
**Gene Size (bp)**	462	1350	2739	540	1062
**Protein size (aa)**	153	449	912	179	353
**M. W (kDa)**	16	48	103	20	37
**pI Value**	6.09	6.20	9.34	8.36	9.62
**VaxiJen score**	0.1642	0.5364	0.5989	0.6058	0.6150
**Allergen non-allergen**	Allergen	Non-Allergen	Non-Allergen	Non-Allergen	Non-Allergen
**Helices <2**	zero	zero	zero	zero	zero
**Estimated half-life**	30hrs	30hrs	30hrs	30hrs	30hrs
**Instability index**	24.62	23.55	38.14	46.99	20.48
**Aliphatic index**	79.87	82.38	71.24	82.40	82.75
**Non-Homologous**	yes	yes	yes	yes	yes

Supplementary Table S2. Nine selected epitopes based on the best scores values (Bibi et al., 2021) [35]

Protein	Epitope	B-Cell Epitopes	T cell epitope	Binding energy score kcal/mol	VaxiJen score	Ic50< 100nm
**Pal OMP P6**	P E1	AACSSSNNDAAGNGAAQTFG	CSSSNNDAA	-5.4	1.4973	31
**Gdh**	G E1	ADVLFGPGKAANAGGVATSG	VLFGPGKAA	-6.2	0.9723	38
	G E2	TTLPMGGAKGGSDFDPKGKS	TTLPMGGAK	-5.1	1.0268	38
	G E3	HVGADTDVPAGDIGVGGREV	DTDVPAGDI	-6.4	1.3879	39
**Tbp1**	**T E1**	**CTNGYESCKKSPKPPAKLST**	**CTNGYESCK**	**-7.2**	**0.9685**	**40**
	T E2	SNPYLYPKPEPYFAGGDLCN	YLYPKPEPY	-7.8	0.4609	40
	**T E3**	**LSKNGTGKGNYGYHNAQNAK**	**GTGKGNYGY**	**-6.8**	**1.3176**	**35**
**PilW**	PW E1	EQAQQQFELALNSPNYYHQA	AQQQFELAL	-5.4	0.7423	41
**Porin**	PorinE1	DAISATGYGKANPVTGATCD	KANVTGAT	-5.8	0.9864	41

Reference:

Bibi, N., Zaidi, N. S., Tahir, M., & Babar, M. M. (2021). Vaccinomics-driven proteome-wide screening of Haemophilus influenzae for the prediction of common putative vaccine candidates. *Can J Microbiol*, *67*(11), 799-812. https://doi.org/10.1139/cjm-2020-0535
